# Functional Specialization in Proline Biosynthesis of Melanoma

**DOI:** 10.1371/journal.pone.0045190

**Published:** 2012-09-14

**Authors:** Jessica De Ingeniis, Boris Ratnikov, Adam D. Richardson, David A. Scott, Pedro Aza-Blanc, Surya K. De, Marat Kazanov, Maurizio Pellecchia, Ze'ev Ronai, Andrei L. Osterman, Jeffrey W. Smith

**Affiliations:** Sanford|Burnham Medical Research Institute, La Jolla, California, United States of America; University of Cantebury, New Zealand

## Abstract

Proline metabolism is linked to hyperprolinemia, schizophrenia, cutis laxa, and cancer. In the latter case, tumor cells tend to rely on proline biosynthesis rather than salvage. Proline is synthesized from either glutamate or ornithine; both are converted to pyrroline-5-carboxylate (P5C), and then to proline via pyrroline-5-carboxylate reductases (PYCRs). Here, the role of three isozymic versions of PYCR was addressed in human melanoma cells by tracking the fate of ^13^C-labeled precursors. Based on these studies we conclude that PYCR1 and PYCR2, which are localized in the mitochondria, are primarily involved in conversion of glutamate to proline. PYCRL, localized in the cytosol, is exclusively linked to the conversion of ornithine to proline. This analysis provides the first clarification of the role of PYCRs to proline biosynthesis.

## Introduction

Since the classic work of Otto Warburg in the 1920s, it has been widely recognized that metabolic rewiring is an essential component of malignant transformation. There is now a renewed interest in cancer metabolism as a potential avenue for diagnosis and treatment [1]. Beyond the increase in glycolysis described by Warburg, tumor cells tend to switch from recycling and salvaging nonessential amino acids to their *de novo* synthesis. Biosynthesis of serine is key to tumor growth [2], and we also observed a strong tendency for breast cancer cells to rely on synthesis rather than salvage of proline [3,4]. In our recent comparative metabolic profiling of melanoma cell lines, we observed increased *de novo* proline synthesis as compared to melanocytes [5]. These observations may relate to the recent finding that the c-Myc activates the biosynthetic branch of proline [6].

**Figure 1:**
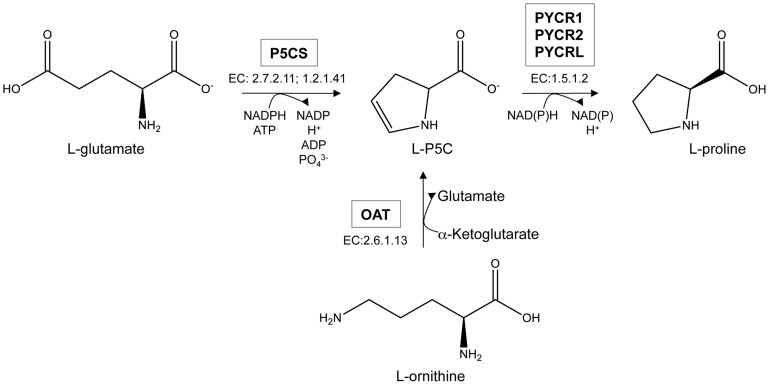
Proline is synthesized via two alternative pathways, from glutamate or ornithine. The enzymes carrying out the reactions shown in the figure are as follows: pyrroline-5-carboxylate synthase (P5CS), which is a fusion of glutamate 5-kinase, (EC 2.7.2.11) and glutamate-5-semialdehyde dehydrogenase (EC 1.2.1.41); ornithine aminotransferase (OAT); pyrroline-5-carboxylate reductase (PYCR), which in humans exists in three isoforms PYCR1, PYCR2 and PYCRL.

Along with salvage, there are two routes to proline: (i) the glutamate route and (ii) the ornithine route [Figure 1]. Both biosynthetic routes converge at pyrroline-5-carboxylate (P5C), the key metabolic intermediate. Glutamate is converted to proline by the sequential action of pyrroline-5-carboxylate synthase (P5CS) and PYCR. Ornithine is converted to proline by the sequential action of ornithine aminotransferase (OAT) and PYCR. Importantly, P5C is so rapidly converted to proline that it is virtually undetectable in cells [7,8]. Consequently, any study aimed at determining whether proline biosynthesis proceeds via convergent pathways must rely on measures of glutamate and ornithine as precursors.

PYCR is typically viewed as a single entity, even though there are three human genes encoding three isozymes: *PYCR1* on chromosome 17q25.3, *PYCR2* at 1q42.13, and *PYCRL* at 8q24.3. PYCR1 (319aa) and PYCR2 (320aa) are very similar (84%), whereas PYCRL is ∼40aa shorter at the C-terminus and is only 45% similar to the other two forms. Of the PYCRs, only PYCR1 has been studied. A defect in this enzyme results in a rare skin disease called Cutis laxa, in which patients present with progeroid features [9]. Functional genetic screens show that PYCR1 is involved in the growth of mammary tumors [2].

Given the paucity of information on PYCR2 and PYCRL, and the lack of information on the role of each PYCR along the two biosynthetic routes to proline, we compared their cellular function and enzymatic properties in melanoma cells lines. The metabolic fate of ^13^C-labeled precursors, combined with gene silencing, allowed us to dissect the contribution of each PYCR to the two alternate routes of proline biosynthesis.

## Materials and Methods

### Cell culture and isotopic labeling

The following melanoma cell lines were used: WM35, Mel501, UACC903, WM793, Lu1205, MeWo, WM1366, WM1346, SBCl2, WM3629 [10,11]. Cells were cultured in DMEM with 10% fetal bovine serum (FBS) and 1% v/v Penicillin/Streptomycin solution (Omega); primary human melanocytes (NEM-LP; Invitrogen) were grown in 254 media supplemented with HMGS (Cascade Biologics). All the cell lines were grown in 5% CO_2_ at 37°C. Cells were labeled in MEM containing 10% dialyzed fetal bovine serum for 8 hr when [U-^13^C] glutamine (1 mM) or [U-^13^C] ornithine (1 mM) were used as isotopic precursors; for 24 hours when [U-^13^C] glucose (2 g/L) was used. Preliminary experiments conducted on cells labeled with [U-^13^C] glutamine (1 mM), established that steady state labeling in downstream metabolites was reached at 6–8 hr [Table S1]. Based on these observations, experiments on the impact of gene silencing were performed feeding the cells with [U-^13^C] glutamine or [U-^13^C] ornithine as precursors. In the case of ornithine, sufficient isotopic labeling in proline could only be generated in the absence of extracellular proline, and in the presence of 1 mM of [U-^13^C] ornithine, even though these conditions are likely not physiologic.

### Quantifying isotopically labeled metabolites with GC-MS

Cells were harvested by trypsinization for 5 min and maintained on ice for all subsequent steps. Cell pellets (1–5×10^6^ cells) were resuspended in 0.6 ml cold (−20°C) 50% methanol (in water) containing 100 µM L-Norvaline (internal standard) and frozen on dry ice. For analysis, pellets were thawed on ice for 10 min, centrifuged at 14,000 rpm, 5 min, 4°C and the methanol extract was divided into two samples and dried down by centrifugation under vacuum. Extracted metabolites were derivatized by addition of 50 µl (20 mg/ml) methoxylamine-hydrochloride (Sigma, in dry pyridine) and incubation for 20 min at 80°C. After cooling, 50 µl *N*-*tert*-butyldimethylsilyl-*N*-methyltrifluoroacetamide (Sigma) was added and samples were re-incubated for 60 min at 80°C followed by centrifugation at 14,000 rpm, 5 min, 4°C. Metabolites separated by GC were fragmented and ionized by electron impact. The mass (mass/charge) of ionized fragments was scanned over the range m/z 150–600. Mass intensity scans were averaged across the time interval in which each metabolite eluted from GC, to provide an average mass spectrum for analysis [5]. The metabolites were quantified based on specific mass ions by METAQUANT [12].

### Calculation of isotopic enrichment and enrichment ratios

Isotopic enrichment is the degree to which isotope appears in a product, and in the case of metabolism it can be used as an indicator of the degree of conversion of precursor into product. Isotopic enrichment in proline was calculated from the proline fragment with mass ≥258. The mass distribution for this fragment was corrected for natural abundance of heavy isotopes using matrix-based probabilistic methods as described [13,14], implemented in Microsoft Excel [15]. Following this correction, the mass distribution (m0, m1, m2, m3, m4, where m0 represents the fraction of this 4-carbons fragment of proline without ^13^C enrichment, m1 represent the presence of one ^13^C carbon in the fragment, m2 the presence of two ^13^C carbons, etc.) was converted to isotopic enrichment per carbon using the following equation:



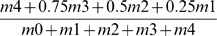



Isotopic enrichment in glutamate and ornithine was calculated in a similar manner. The fraction of proline derived from either glutamate or ornithine is expressed as the isotopic enrichment ratio (e.g. isotopic enrichment of proline/isotopic enrichment of glutamate).

### Immune-blotting

Cell pellets were extracted with lysis buffer (25 mM TRIS pH 8, 150 mM NaCl, 1 mM CaCl_2_, 1% triton X-100, 1 mM PMSF and protease inhibitor cocktail (Sigma)). Proteins (20 µg) were separated on SDS-PAGE and blotted to PVDF membrane, and probed with the following antibodies: PYCR1, H00005831-B01P (Abnova); PYCR2, SAB2101919 (Sigma); PYCRL, H00065263-M01 (Abnova); P5CS, H00005832-M01 (Abnova); OAT, PO4181 (Epitomics).

### Gene Silencing with siRNA

For transfection with siRNA, cells were seeded at 250,000 cells per 10 cm culture dish in MEM (Cellgro 15-010: 1 g/L glucose, w/o glutamine) with 10% v/v dialyzed fetal bovine serum, 2 mM L-glutamine, 1% v/v Penicillin/Streptomycin solution (Omega), 1% v/v MEM vitamins (Irvine Scientific) and 1% of non-essential amino acids. Cells were transfected with siRNA the day after seeding using RNAimax (Invitrogen), and maintained in the same medium for an additional 72 hr prior to isotopic labeling. L-Proline (Sigma) was also added as indicated. Quantitative PCR was used to measure the extent of knock down of mRNA. Total RNA was isolated using a RNAsy mini kit (Qiagen) and reverse transcribed using a SuperScript III First-Strand Synthesis SuperMix for qRT-PCR (Invitrogen). Specific primers used for qPCR were as follows: PYCR1 fw 5′-tttctgctctcaggaagatg-3′; PYCR1 rev 5′-accacaatgtgtctgtcctc-3′; PYCR2 fw 5′-tccctcgctgagggggttcgt-3′; PYCR2 rev 5′-ccatcttcctgagcgcggacacc-3′; PYCRL fw 5′-cccagaccctgctgggggacg-3′; PYCRL rev 5′-ctccacggcgctcatggtgg-3′; P5CS fw 5′-catgagaacctccctattcc-3′; P5CS rev 5′-atccaggtacactttccaa-3′. Human cyclophilin A was used as a control. The reaction mixture was denatured at 95°C for 10 min, followed by 40 cycles of 95°C for 30 s, annealing at 56°C for 60 s and extension at 72°C for 30 s. Reactions were performed using the SYBRGreenER Universal qPCR Mix (Invitrogen) and run on an MX3000P qPCR cycler (Stratagene). The specificity of the products was verified by melting curves analysis. The target mRNA levels were normalized to the level of mRNA encoding cyclophilin A. In cases where PYCR1 was silenced with siRNA we observed a reduction in cell viability of ∼50%, but changes in isotopic enrichment in proline from glutamate could not be attributed to this phenomenon because isotopic enrichment in other metabolites were not affected [Table S2]. A similar, but smaller reduction in cell proliferation was observed when PYCRL was silenced with siRNA, but this had no effect on other metabolites [Table S2].

### Sub-cellular localization of PYCRs

Cells from a confluent 15 cm dish were washed in PBS and resuspended in isotonic buffer HM (10 mM Hepes, pH 7.4, 250 mM mannitol, 10 mM KCl, 5 mM MgCl2, 1 mM EGTA), washed again and homogenized with 100 strokes and a B-type Pestle (cells were checked with trypan blue by microscope during homogenization). The suspension was centrifuged at 2,500 rpm for 5 min twice. The supernatant was centrifuged again at 10,000 rpm, 10 min, 4°C to separate the mitochondria (pellet) from the cytoplasm fraction (supernatant). The mitochondria were washed twice with HM buffer whereas the cytosolic fraction was clarified by centrifugation at 14000 rpm, 30 min, 4°C. Mitochondria were lysed in the lysis buffer (see immune-blot section) and total proteins were quantified. PYCRs were detected in each fraction by immunoblotting as above. IKBα (Cell Signaling) was used as a cytosolic marker and VDAC1 (Santa Cruz Biotechnology Inc., USA) as a mitochondrial marker.

### Expression of recombinant PYCRs

PYCR2 and PYCRL genes were amplified from clones obtained from Open Biosystem. PYCR1 was amplified from a plasmid kindly donated by Dr. Z. Meng (Tsinghua University, Beijing). The three genes were amplified by PCR with the following primers: PYCR1 fw and PYCR2 fw 5′-acacacggatccatgagcgtgggcttc-3′; PYCR1 rev 5′-taacaactcgagtcaatccttgcccg-3′; PYCR2 rev 5′-gtgccactcgagttagtccttcttgcctcc-3′; PYCRL fw 5′-acacacggatccatggcagctgcgg-3′; PYCRL rev 5′-gtgccactcgagctactttctgctgagctcc-3′. The three genes were cloned into pSMT3 vector (N-terminal 6xHis_SUMO tag) [16] using BamHI/XhoI cloning sites. The resulting constructs were transformed in *E. coli* BL21/DE3 cells and protein expression was carried out at 22°C in the presence of 0.2 mM IPTG for 16 hours. The recombinant soluble protein supernatant was purified using Ni-NTA (Qiagen) affinity chromatography. The SUMO tag was removed by 18 hr incubation with 1∶100 (w/w) ULP-1. The cleaved SUMO His tag fusion protein and the ULP protease were removed by Ni-NTA affinity chromatography. The purity of each recombinant enzyme was gauged by SDS-PAGE.

### Enzymatic characterization of PYCRs

Enzymatic assays were performed by continuous monitoring of consumption of NADH or NADPH at 340 nm or 380 nm in the course of conversion of Δ^1^-Pyrroline-5-Carboxylic Acid (P5C) to proline. Concentrations of NADH and NADPH (Sigma) were determined from light absorbance values at 340 nm using molar extinction coefficient of 6,200 M^−1^cm^−1^ or at 380 nm using molar extinction coefficient of 1,314 M^−1^cm^−1^. P5C was prepared from (2*S*)-di-*tert*-butyl 5-hydroxypyrrolidine-1,2-dicarboxylate synthesized as described [17] by hydrolysis of 60 mg of slurry in 0.5 mL 1 N HCl for 24 hrs at ambient temperature with vigorous stirring. The P5C content was measured using 2-Aminobenzaldehyde (Sigma) as described [18]. The hydrolyzed P5C was diluted 5-fold in deionized water to yield 0.2 N concentration of HCl. P5C concentration in a typical preparation ranged from 38 to 42 mM. Diluted P5C was stored at −80°C and tested for P5C content prior to enzymatic analysis. Assays were carried out in 300 mM Tris, pH 8.0 containing 0.01% Brij 35 at 37°C. Apparent kinetic constants for the substrate and co-factors were measured at saturating concentrations of the co-factors and substrate respectively. P5C concentrations used for kinetic analysis ranged from 0 to 10 mM. NAD(P)H concentrations used for kinetic analysis ranged from 0 to 5 mM. Determination of the apparent inhibition constants of PYCRs by proline were performed at saturating concentrations of co-factors by measuring changes in apparent Km values for P5C at different concentrations of inhibitor. All measurements were performed at three different concentrations of each enzyme (PYCR1 – 25, 12.5 and 6.25 nM, PYCR2 – 12.5, 6.25 and 3.25 nM, and PYCRL – 50, 25 and 12.5 nM). Data fitting and determination of kinetic parameters were performed using Prism (GraphPad).

## Results

### PYCRs are up-regulated in melanoma cells

**Figure 2:**
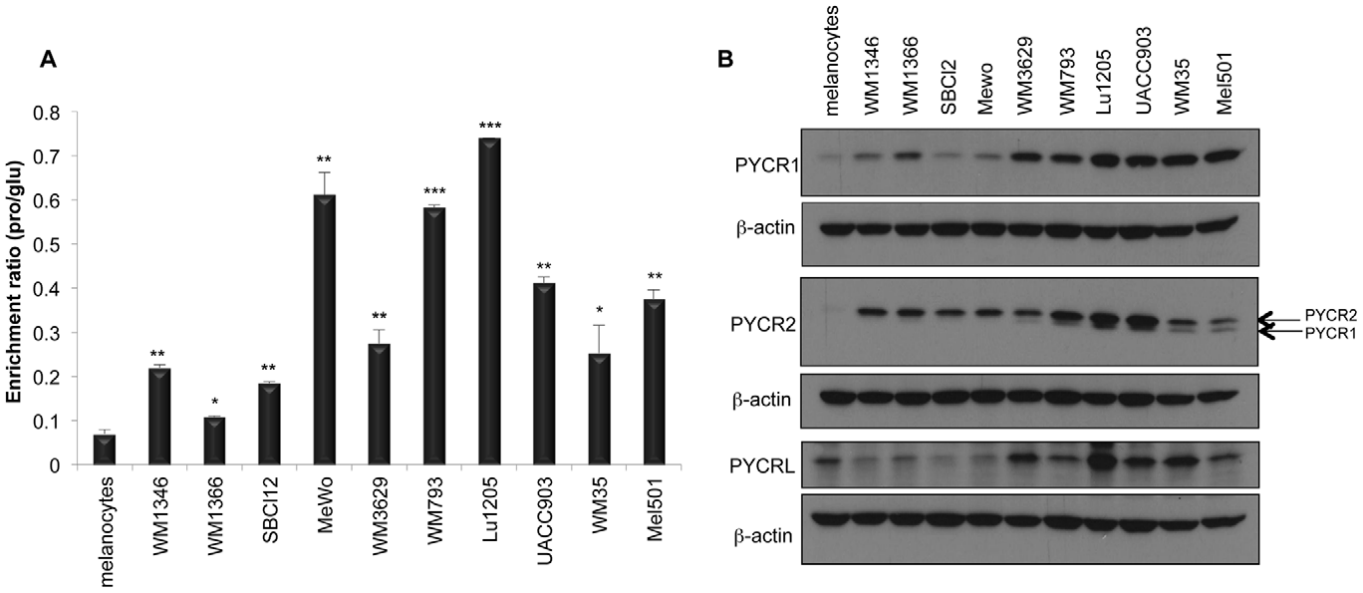
*De novo* proline biosynthesis and expression of PYCRs are upregulated in melanoma compared to primary melanocytes. (A) Melanocytes and a panel of ten melanoma cell lines were labeled with [U^13^C]-Glucose (2 g/L) in the presence of 0.3 mM exogenous proline for 24 hours and the enrichment ratio (pro/glu) was measured by GC-MS as described in Materials and Methods. Error bars represent standard deviations of biological duplicates. Statistical significance of differences observed between melanocytes and melanoma cell lines was determined using Student's t-test (*p<0.05; **p<0.01; ***p<0.001) (B) Western blotting was used to compare expression levels of each PYCR between melanocytes and melanoma cells. Specificities of the antibodies used are listed on the left of each panel. The antibody against PYCR2 shows some cross-reactivity with PYCR1 (arrows). β-actin was used as loading control.

Experiments were conducted to compare biogenesis of proline in ten melanoma cell lines and melanocytes at physiologic concentrations of exogenous proline (0.3 mM). Cells were fed [U-^13^C] glucose and the isotopic enrichment in proline and glutamate was calculated as described in Material and Methods. In the melanoma cell lines the fraction of proline derived from glutamate, indicated as isotopic enrichment ratio (pro/glu), was three to ten-fold higher than in melanocytes [Figure 2A]. However, the salvage of exogenous proline from the medium was not impaired in the melanoma cell lines.

Given this observation we compared the expression of the PYCRs in melanoma cells with Western blots [Figure 2B]. PYCR1 and PYCR2 are abundant in melanoma cells but not detected in melanocytes. PYCRL is expressed to some degree in melanocytes but is more expressed in some melanoma cell lines. Expression of P5CS, the enzyme that converts glutamate to P5C, is also higher in melanoma than in melanocytes [Figure S1]. However, OAT, which can generate P5C from ornithine, is expressed at similar levels in melanoma and melanocytes [Figure S1].

### The individual PYCRs function along distinct biosynthetic routes

Gene silencing experiments combined with ^13^C labeling were conducted to measure the relative contribution of each PYCR along the different biosynthetic routes to proline [Figure 1]. If a silenced enzyme functions along a pathway contributing the isotopically labeled precursor, then isotopic enrichment in the product (proline) relative to the precursor (glutamate) will decrease. However, when the targeted enzyme functions primarily along a biosynthetic pathway contributing non-isotopically-enriched carbon (e.g. from ornithine), different outcomes are possible. In the simplest case, siRNA knockdown will decrease flux of ^12^C toward proline, and thereby cause an *increase* in the isotopic enrichment in proline from glutamate (pro/glu). It is also conceivable that the uptake of ^12^C-Pro by salvage could increase to compensate for knockdown of biosynthesis of proline from ornithine. In such case, the effects of knockdown of biosynthesis from ornithine on the enrichment ratio could be masked. The outcomes of the knockdown experiments described below were interpreted with this framework in mind.

**Figure 3:**
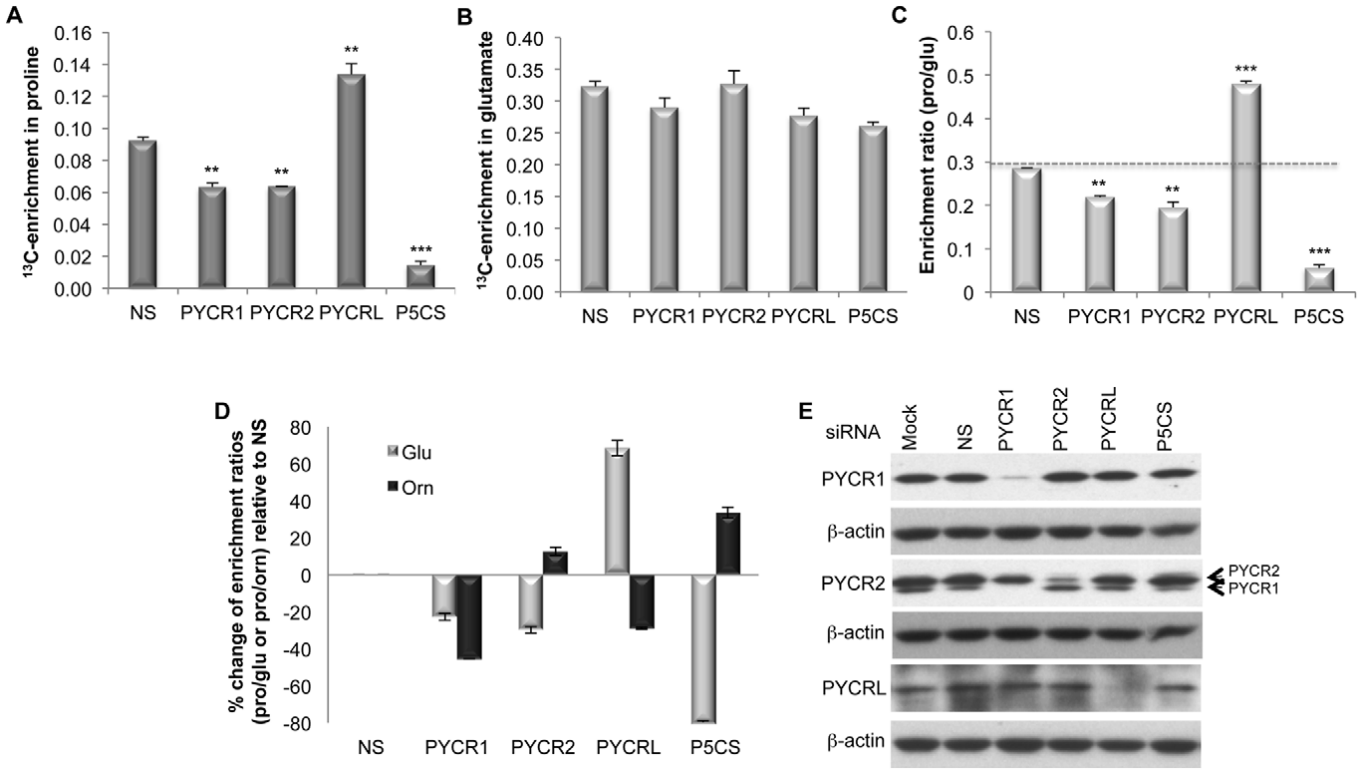
Individual PYCRs contribute to alternative pathways of proline biosynthesis. Lu1205 cells were labeled with [U-^13^C] glutamine (1 mM) for 8 hours in the presence of 0.5 mM proline in the medium and with [U-^13^C] ornithine (1 mM) for 8 hours in the absence of exogenous proline, following silencing of PYCR1, PYCR2, PYCRL or P5CS. Error bars represent standard deviations of duplicate measurements from two independent experiments. (A) ^13^C-enrichment in proline when cells were labeled with [U-^13^C] glutamine measured as described in Material and Methods. Statistical significance of differences observed between specific and non specific (NS) knockdown was determined using Student's t-test (*p<0.05; **p<0.01; ***p<0.001). (B) ^13^C-enrichment in glutamate when cells were labeled with [U-^13^C] glutamine measured as described in Material and Methods. (C) Fraction of proline generated from glutamate expressed as enrichment ratio (pro/glu) determined as described in Material and Methods. Statistical significance of differences observed between specific and non specific (NS) knockdown was determined using Student's t-test (*p<0.05; **p<0.01; ***p<0.001). (D) Comparison between the changes of enrichment ratios (pro/glu and pro/orn) relative to non-specific siRNA (NS) control, expressed as %. (E) Western blot analysis was used to determine protein expression levels. Specificities of the antibodies used are indicated on the left of each panel. Antibodies against PYCR2 cross-react with PYCR1 (indicated by arrows). β-actin was used as loading control.

Results from experiments conducted in Lu1205 cells, which present a high expression of all three PYCRs and have aggressive metastatic properties 19,20] are shown as an example [Figure 3], but similar results were obtained in three other melanoma cell lines [Figure S2]. The knockdown of P5CS is used as a positive control because it produces the substrate of PYCRs, P5C, from glutamate. When [U-^13^C] glutamine was used as metabolic tracer, the gene knockdown affected the isotopic enrichment in proline [Figure 3A, Table S3] but not the isotopic enrichment in glutamate [Figure 3B, Table S4]. Knockdown of P5CS decreased the fraction of proline derived from glutamate, referred as the isotopic enrichment ratio (pro/glu), by 80%, validating the approach and indicating the dynamic range of possible knockdown effects [Figure 3C,D; Table S5]. Knockdown of PYCR1 and PYCR2 reduced isotopic enrichment ratio (pro/glu) by 24% and 31%, respectively, indicating that they both contribute to the biosynthesis of proline from glutamate in a similar manner. However, knockdown of PYCRL led to a 66% increase in isotopic enrichment of proline from glutamate compared to the control [Figure 3C, D; Table S5].

Similar experiments were done with [U-^13^C] ornithine as the metabolic precursor. Since P5CS is known to function only along the pathway from glutamate to proline, its silencing increased the isotopic enrichment ratio (pro/orn) as expected [Figure 3D; Table S6]. Similarly, silencing of PYCR2 increased the isotopic enrichment ratio (pro/orn), confirming that this enzyme is predominantly involved in the glutamate pathway. However, silencing of either PYCR1 or PYCRL decreased the isotopic enrichment ratio (pro/orn) by 51% and 34%, respectively [Figure 3D; Table S6], confirming the unique function of PYCRL along the ornithine route and also indicating some contribution of PYCR1 to this route (at least under the condition of low proline and high ornithine level in the culture medium).

### PYCRs are expressed in distinct cellular compartments

**Figure 4:**
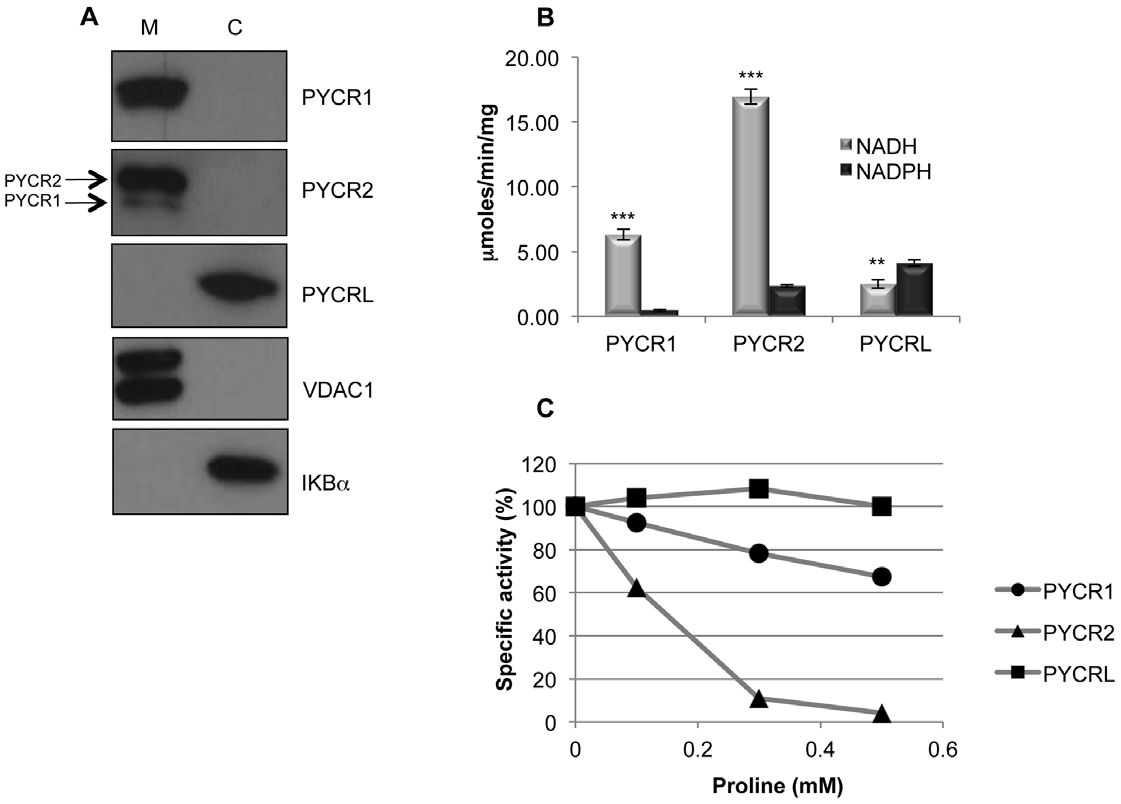
PYCRs are characterized by differential subcellular localization, co-factor preference and sensitivity to product inhibition. (A) Presence of each PYCR in mitochondrial (left) (5 µg protein) and cytosolic (right) (30 µg protein) fractions was determined by Western blotting following sub-cellular fractionation of Lu1205 cells as described in Materials and Methods. VDAC1 and IKBα were used as mitochondrial and cytosolic markers, respectively. (B) The efficiency of conversion of P5C (0.1 mM) to proline by each recombinant PYCR in the presence of NADH vs. NADPH (each at 0.1 mM) as cofactors was measured as described in Materials and Methods. Error bars represent standard deviations of three measurements obtained at different enzyme concentrations. Statistical significance of differences observed between the two co-factors was determined using Student's t-test (*p<0.05; **p<0.01; ***p<0.001). (C) Sensitivity to product inhibition of each recombinant PYCR was determined by measuring their specific activities across a concentration range of proline (0.1–0.5 mM) in the presence of 0.1 mM P5C and NADH. Triplicate measurements were made and the standard deviations were always less than 5%.

To gain further insight into the functional specialization of the PYCRs, we determined their subcellular localization. This was accomplished with Western blotting using standard approaches to fractionate Lu1205 cells into mitochondrial and cytosolic fractions. IKBα, a known cytosolic protein, and the voltage-dependent anion channel (VDAC1), a known mitochondrial protein, were used as markers. PYCR1 and PYCR2 are strictly associated with mitochondria, but PYCRL is found only in the cytoplasm [Figure 4A]. Clearly then, PYCRL is partitioned away from the other enzymes of the P5C sub-network, which are located in the mitochondria.

### PYCRs exhibit distinct enzymatic properties

The enzymatic properties of recombinant forms of each PYCR were compared. Of the three PYCRs, PYCR1 was the only one enzymatically characterized prior to this study using proline as substrate (reverse direction) [21]. We measured the enzymatic activity in the reductive direction (forward) from P5C to proline. Activity was gauged by measuring the conversion of either NADH to NAD^+^ or NADPH to NADP^+^. At physiologic concentrations of P5C (substrate) and co-factors, PYCR1 and PYCR2 have higher specific activity in the presence of NADH, consistent with their localization in the mitochondria. Not surprisingly, PYCRL is more efficient with NADPH as a cofactor [Figure 4B]. This difference in cofactor preference can be attributed to the fact that PYCRL has much lower Km for P5C (∼10-fold lower) in the presence of NADPH [Table S7]. This observation is supported by the evolution analysis of the proteins, which show that PYCRL is more closely related to the ancestral form of PYCR by exhibiting higher similarity with the bacterial enzyme that notably prefers NADPH [22]. Only in higher eukaryotes there was a diversification of a more recent form of PYCR that finally split into PYCR1 and PYCR2.

Another key difference among the PYCRs is their sensitivity to product inhibition [Figure 4C]. PYCRL is the least sensitive to inhibition by proline (Ki_app_ = 8 mM). PYCR1 (Ki_app_ = 0.6 mM) and PYCR2 (Ki_app_ = 0.1 mM) (Table S7) are inhibited in the physiologic range of proline. PYCR2 is the most sensitive, losing 90% of its activity at 0.3 mM proline.

### Biogenesis of proline is regulated by its extracellular concentration

The sensitivity of PYCR1 and PYCR2 to product inhibition (by proline) prompted us to determine the impact of different concentrations of extracellular proline (0–0.5 mM) on its cellular biogenesis. Proline synthesized through the glutamate pathway, where both PYCR1 and PYCR2 function, decreased as extracellular proline concentration increased. Since PYCRL is uniquely responsible for the biosynthesis of proline from ornithine in physiological conditions, the contribution of the ornithine route was calculated from the isotopic enrichment ratio (pro/glu) when PYCRL is knocked down [Table 1, Table S5]. The contribution of proline salvage was determined as the residual fraction from the sum of the three convergent pathways (equal to 1). As a result, proline synthesized through the ornithine route increased as extracellular proline concentration increased. These observations are consistent with the finding that proline inhibits pure recombinant enzymes PYCR1 and PYCR2, but not PYCRL, and suggests a compensatory mechanism of the ornithine route to ensure a certain rate of proline biosynthesis. At physiologically relevant concentrations of proline, the contribution of each biosynthetic route and the salvage route are comparable [Table 1].

**Table 1:** Relative contribution of convergent pathways to proline is regulated by extracellular proline.

Pro (mM)	0	0.1	0.3	0.5
**Glutamate synthesis route**	0.95	0.76	0.46	0.29
**Ornithine synthesis route**	0.05	0.07	0.19	0.40
**Proline salvage**	0	0.17	0.35	0.31

Lu1205 cells were labeled with [U-^13^C] glutamine (1 mM) for 8 hr in the presence of different concentrations of proline in the medium (0, 0.1, 0.3, 0.5 mM). The isotopic enrichment in glutamate and proline was calculated, and the fraction of proline derived from glutamate is indicated by the ratio of these two values. Since PYCRL is the primary enzyme for proline biosynthesis from ornithine, the fraction of proline synthesized via this route was determined from the same experiment performed in cells where PYCRL was knocked down. In this case the ornithine route is contributing unlabeled carbon to the proline pool, so knockdown of PYCRL (≥90%) reduces the amount of unlabeled carbon in the proline pool, and therefore actually *increases* the enrichment ratio (pro/glu) [Table S5]. Therefore the contribution of carbon from the ornithine route is calculated by determining how much unlabeled carbon must be lost (via PYCRL knockdown) to increase the enrichment ratio (pro/glu) as indicated [Table S5]. The formula for this value is:

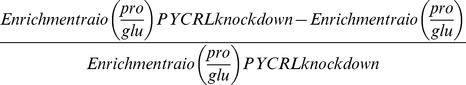

Considering the sum of the three convergent pathways equal to 1, the contribution of proline salvage was determined as the residual from the total.

## Discussion

**Figure 5:**
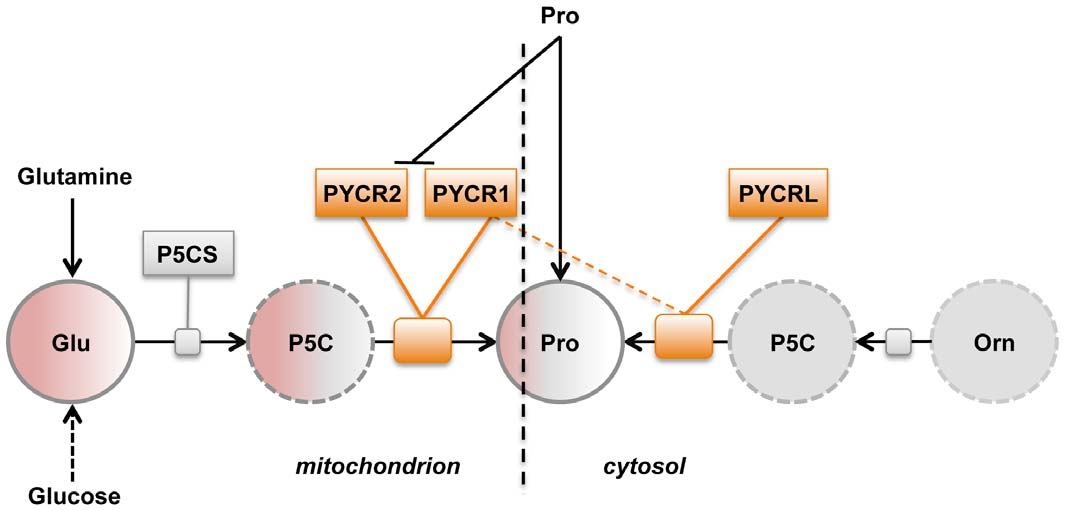
Model of proline biogenesis in melanoma . The cartoon presents a model that provides the simplest interpretation of the findings presented here. Sub-cellular localization and the roles of the three PYCRs in proline biosynthesis are shown. PYCR1 and PYCR2 are located in the mitochondria, and both have access to the pool of P5C generated from glutamate by P5CS. Under certain conditions (such as high concentration of ornithine and low proline in the medium) PYCR1 has access to a P5C pool generated in the cytoplasm (dotted line). In contrast, PYCRL primarily uses a cytosolic pool of P5C. Salvage of proline from extracellular sources is also shown as well as its inhibitory effects on PYCR1 and PYCR2. Abbreviations: Glutamate (Glu), Pyrroline-5-Carboxylate (P5C), Proline (Pro), Ornithine (Orn), Pyrroline-5-Carboxylate Reductase (PYCR), Pyrroline-5-Carboxylate Synthase (P5CS).

Here we show that the three PYCRs make distinct contributions to proline biosynthesis in melanoma cells. PYCR1 contributes primarily to production of proline from glutamate, but under some conditions (no extracellular proline and high ornithine) it can also function along the ornithine route. However, it is unlikely that in these cell line PYCR1 participates in this route in physiological conditions. On the other hand, we cannot exclude the possibility that it may happen in other cell lines. PYCR2 is used exclusively for biosynthesis of proline from glutamate, and PYCRL participates only in production of proline from ornithine. Based on our findings, we propose a working model of proline biosynthesis [Figure 5]. The model illustrates the contribution of each PYCR to the two biosynthetic routes to proline in the context of the sub-cellular localization and enzymatic properties of each enzyme.

One implication of this study, which is illustrated in the working model, is that P5C exists in separate unmixed pools. Although the steady-state level of P5C is too low to be measured directly, the inference of separate P5C pools follows from the results of gene silencing experiments. Thus, knockdown of PYCRL reduces the isotopic enrichment in proline from ornithine, but not from glutamate. However, if P5C, which is the common intermediate along both routes, existed as a single pool, then knockdown of PYCRL would alter the isotopic enrichment in proline in the same way from both precursors. This observation is consistent with the idea that the observed P5C pool separation may reflect its *channeling* within multi-enzyme complexes that contain the PYCRs, as has been observed in other systems [9,23,24]. The fact that PYCRL functions exclusively in the cytoplasm to convert P5C to proline, raises questions about how P5C is generated in the cytosol. This remains an open issue because OAT is in the mitochondrial matrix, but we cannot exclude the possibility that some form of OAT exists in the cytosol and is coupled to PYCRL.

The PYCRs are functionally tied to proline dehydrogenase (PRODH), which catalyzes the conversion of proline to P5C in the mitochondria. In essence then, PRODH reverses the action of PYCR activity. Superoxide generated by PRODH makes cells more sensitive to stress [25,26,27,28], probably explaining the pro-apoptotic and tumor suppressor function of PRODH [29]. Given their mitochondrial localization, PYCR1 and PYCR2 could potentially partner with PRODH in this process. However, PRODH is generally down-regulated in tumors [29], and without this reaction the end point of PYCR activity is probably production of proline. Since our results show that PYCR2 is inhibited by proline at the lower end of the physiologic concentration range, PYCR1 is likely to be the dominant enzyme in proline biosynthesis.

Three enzymes involved in the biosynthetic route from glutamate to proline (P5CS, PYCR1 and PYCR2) are up-regulated in melanoma compared to melanocytes. The ^13^C enrichment in proline from glutamate is also much higher in melanoma cell lines. Together these observations point to a potential role for this biosynthetic route in progression of melanoma. This is not the first association between PYCR and tumors. A recent study found that expression of PYCR1 is up-regulated in prostate cancer [30]. Another study found that PYCR1 is causally linked to growth of breast cancer [2]. The role of PYCR1 in cancer is not entirely clear, but it probably relates to the biosynthesis of proline and potentially production of collagen for the extracellular matrix. The latter possibility may be especially relevant to the tumor extracellular matrix because individuals with mutations in PYCR1 have abnormal collagen fibrils [31]. While it is not up-regulated in all melanoma cell lines, PYCRL could still play an important role in cell growth. PYCRL could couple to the pentose phosphate pathway (PPP) because it is localized in the cytosol, and because it produces NADP, a key cofactor for the PPP. This idea is supported by the fact that addition of extracellular P5C, which is the substrate for PYCRs, is immediately converted to proline in cells and activates the PPP [7,8].

This study also illustrates an important technical point on the silencing of metabolic enzymes with siRNA. In the case of these targets, one cannot always expect the knockdown with siRNA to produce a proportional inhibition of ^13^C enrichment from a substrate to a product. Indeed, the efficiency of gene knockdown by siRNA is usually 85–95%, so in almost all cases some enzyme is expressed. In fact, others have also observed less than complete inhibition of isotopic enrichment of a product from a substrate when silencing metabolic enzymes with shRNA [2,32]. If the enzyme in question is not the rate-limiting step, then even 5-10% remaining enzyme could support substantial conversion of a substrate into a product. This is likely to be part of the reason that knockdown of P5CS reduces the isotopic enrichment in proline from glutamate by nearly 90%, but knockdown of PYCRs fails to reach this degree. Furthermore, in this instance, where two PYCRs function along a particular path, knockdown of one isozyme is expected to only partially reduce the ^13^C enrichment in proline from the respective precursor.

In summary, the biogenesis of proline is regulated by three PYCRs that have distinct sub-cellular localization and enzymatic properties. PYCR1 and PYCR2 are localized in the mitochondria, and primarily involved in the conversion of glutamate to proline, and are subject to product inhibition. PYCRL is a cytoplasmic enzyme, exclusively involved in conversion or ornithine to proline. This enzyme is not inhibited by proline. Now that we have established the role of each PYCR in proline biosynthesis, and have illuminated distinctions in their enzymatic properties, it will be possible to probe their role in melanoma and other diseases in an informed manner.

## Supporting Information

Figure S1 **Melanoma cells express higher levels of P5CS but similar levels of OAT compared to primary melanocytes.** Expression of P5CS and OAT in melanocytes relative to melanoma cells represented by a panel of ten cell lines was determined by Western blotting. Specificities of the antibodies used are indicated on the left of each panel. β-actin was used as loading control.
(TIFF)

Figure S2 **Effect of PYCRs silencing in UACC903, WM1346 and WM1366 cells.** The production of proline from glutamate, expressed as the enrichment ratio (pro/glu), was measured in cells labeled for 8 hr with [U-^13^C] glutamine (1 mM) in the presence of 0.3 mM of proline in the medium. Error bars represent standard deviations of biological duplicate.
(TIFF)

Table S1 **^13^C enrichment in proline and glutamate in Lu1205 cells at different time points.** Cells were fed with [U-^13^C] glutamine in the presence of 0.3 mM of exogenous proline. Measurements of isotopic enrichment in proline and glutamate were made 2, 4, 6 and 8 hr after labeling. Results represent technical duplicates and standard deviations are less than 10%.
(DOCX)

Table S2 **Knockdown of PYCRs is without effect on isotopic enrichment in TCA cycle metabolites.** Lu1205 cells were fed with [U-^13^C] glutamine in the presence of 0.5 mM of exogenous proline and isotopic enrichment was calculated after 8 hr of labeling. Data represent average of biological duplicates and standard deviations are less than 5%.
(DOCX)

Table S3
**^13^C enrichment in proline in Lu1205 cells labeled with [U-^13^C] glutamine (8 h) in the presence of 0.5 mM of proline in the media.** m0 is m/z 258 fragment ion, m1 is m/z 259, etc. Enrichment in proline is calculated with the following formula: 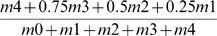
Data represent average of two biological replicates and standard deviations are less than 5%.(DOCX)

Table S4
**^13^C enrichment in glutamate in Lu1205 cells labeled with [U-^13^C] glutamine (8 h) in the presence of 0.5 mM of proline in the media.** m0 is m/z 330 fragment ion, m1 is m/z 331, etc. Enrichment in glutamate is calculated with the following formula: 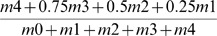 Data represent average of two biological replicates and standard deviations are less that 5%.(DOCX)

Table S5 **Relative contribution of PYCRs to glutamate pathway**. (A) Fraction of proline derived from glutamate expressed as the ratio of ^13^C enrichment of proline (product) over ^13^C enrichment of glutamate (precursor), measured upon silencing of PYCR1, PYCR2, PYCRL and P5CS. Lu1205 cells were labeled with [U-^13^C] glutamine (1 mM) for 8 h in the presence of 0.1, 0.3 and 0.5 mM of proline in the medium. (B) The same data are presented as % of change relative to non-specific siRNA (NS) control. Data are representative of two biological replicates and standard deviations are less than 5%. At 0.3 and 0.5 mM of exogenous proline, the data are consistent and show the same trend (as for PYCRL); at low concentration of proline (0.1 mM) there is not enough contribution of the salvage pathway to allow appreciable differences compared to the NS.
(DOCX)

Table S6 **Relative contribution of PYCRs to ornithine pathway**. (A) Fraction of proline derived from ornithine expressed as the ratio of ^13^C enrichment of proline (product) over ^13^C enrichment of ornithine (precursor), measured upon silencing of PYCR1, PYCR2, PYCRL and P5CS. Lu1205 cells were labeled with [U-^13^C] ornithine (1 mM) for 8 h in the absence of proline in the medium. (B) The same data are presented as % of change relative to non-specific siRNA (NS) control. Data are representative of two biological replicates and standard deviations are less than 5%.
(DOCX)

Table S7 **Kinetic characterization of recombinant PYCRs.** Apparent kinetic parameters of human PYCRs with respect to substrate (A) and cofactors (B) were determined by monitoring the turnover of co-factors (NADH or NADPH). Results from steady-state kinetics experiments, obtained at saturating concentrations of cofactors*** and substrate*** (P5C) were graphed and fit with non-linear regression**. Apparent catalytic efficiencies of human PYCRs with respect to P5C and cofactors (C) were calculated using the apparent kcat and Km values presented in A and B. Apparent constants of PYCR inhibition by proline at saturating concentrations of co-factors (D) (1 mM NADH for PYCR1 and PYCR2 and 1.25 mM NADPH for PYCRL) were determined by fitting the steady-state kinetics data of P5C conversion to proline at varying concentrations of inhibitor with competitive inhibition equation.
(DOCX)

## References

[1] Vander Heiden MG (2011) Targeting cancer metabolism: a therapeutic window opens. Nat Rev Drug Discov 10: 671–684. 21878982 10.1038/nrd3504

[2] Possemato R, Marks KM, Shaul YD, Pacold ME, Kim D, et al (2011) Functional genomics reveal that the serine synthesis pathway is essential in breast cancer. Nature 476: 346–350. 21760589 10.1038/nature10350 PMC3353325

[3] Knowles LM, Smith JW (2007) Genome-wide changes accompanying knockdown of fatty acid synthase in breast cancer. BMC Genomics 8: 168. 17565694 10.1186/1471-2164-8-168 PMC1913522

[4] Richardson AD, Yang C, Osterman A, Smith JW (2008) Central carbon metabolism in the progression of mammary carcinoma. Breast Cancer Res Treat 110: 297–307. 17879159 10.1007/s10549-007-9732-3 PMC2440942

[5] Scott DA, Richardson AD, Filipp FV, Knutzen CA, Chiang GG, et al.. (2011) Comparative metabolic flux profiling of melanoma cell lines: beyond the Warburg effect. J Biol Chem. 10.1074/jbc.M111.282046 PMC3234981 21998308

[6] Liu W, Le A, Hancock C, Lane AN, Dang CV, et al (2012) Reprogramming of proline and glutamine metabolism contributes to the proliferative and metabolic responses regulated by oncogenic transcription factor c-MYC. Proc Natl Acad Sci U S A 109: 8983–8988. 22615405 10.1073/pnas.1203244109 PMC3384197

[7] Mixson AJ, Phang JM (1988) The uptake of pyrroline 5-carboxylate. Group translocation mediating the transfer of reducing-oxidizing potential. J Biol Chem 263: 10720–10724. 3392037

[8] Boer P, Sperling O (1991) Stimulation of ribose-5-phosphate and 5-phosphoribosyl-1-pyrophosphate generation by pyrroline-5-carboxylate in mouse liver in vivo: evidence for a regulatory role of ribose-5-phosphate availability in nucleotide synthesis. Biochem Med Metab Biol 46: 28–32. 1718342 10.1016/0885-4505(91)90047-o

[9] Reversade B, Escande-Beillard N, Dimopoulou A, Fischer B, Chng SC, et al (2009) Mutations in PYCR1 cause cutis laxa with progeroid features. Nat Genet 41: 1016–1021. 19648921 10.1038/ng.413

[10] Smalley KS, Contractor R, Nguyen TK, Xiao M, Edwards R, et al (2008) Identification of a novel subgroup of melanomas with KIT/cyclin-dependent kinase-4 overexpression. Cancer research 68: 5743–5752. 18632627 10.1158/0008-5472.CAN-08-0235 PMC2615688

[11] Shah M, Bhoumik A, Goel V, Dewing A, Breitwieser W, et al (2010) A role for ATF2 in regulating MITF and melanoma development. PLoS genetics 6: e1001258. 21203491 10.1371/journal.pgen.1001258 PMC3009656

[12] Bunk B, Kucklick M, Jonas R, Munch R, Schobert M, et al (2006) MetaQuant: a tool for the automatic quantification of GC/MS-based metabolome data. Bioinformatics 22: 2962–2965. 17046977 10.1093/bioinformatics/btl526

[13] Nanchen A, Fuhrer T, Sauer U (2007) Determination of metabolic flux ratios from 13C-experiments and gas chromatography-mass spectrometry data: protocol and principles. Methods Mol Biol 358: 177–197. 17035687 10.1007/978-1-59745-244-1_11

[14] van Winden WA, Wittmann C, Heinzle E, Heijnen JJ (2002) Correcting mass isotopomer distributions for naturally occurring isotopes. Biotechnol Bioeng 80: 477–479. 12325156 10.1002/bit.10393

[15] Portnoy VA, Scott DA, Lewis NE, Tarasova Y, Osterman AL, et al (2010) Deletion of genes encoding cytochrome oxidases and quinol monooxygenase blocks the aerobic-anaerobic shift in Escherichia coli K-12 MG1655. Appl Environ Microbiol 76: 6529–6540. 20709841 10.1128/AEM.01178-10 PMC2950451

[16] Mossessova E, Lima CD (2000) Ulp1-SUMO crystal structure and genetic analysis reveal conserved interactions and a regulatory element essential for cell growth in yeast. Mol Cell 5: 865–876. 10882122 10.1016/s1097-2765(00)80326-3

[17] Gerratana B, Stapon A, Townsend CA (2003) Inhibition and alternate substrate studies on the mechanism of carbapenam synthetase from Erwinia carotovora. Biochemistry 42: 7836–7847. 12820893 10.1021/bi034361d

[18] Williams I, Frank L (1975) Improved chemical synthesis and enzymatic assay of delta-1-pyrroline-5-carboxylic acid. Anal Biochem 64: 85–97. 166569 10.1016/0003-2697(75)90408-x

[19] Satyamoorthy K, Li G, Gerrero MR, Brose MS, Volpe P, et al (2003) Constitutive mitogen-activated protein kinase activation in melanoma is mediated by both BRAF mutations and autocrine growth factor stimulation. Cancer Res 63: 756–759. 12591721

[20] Krasilnikov M, Ivanov VN, Dong J, Ronai Z (2003) ERK and PI3K negatively regulate STAT-transcriptional activities in human melanoma cells: implications towards sensitization to apoptosis. Oncogene 22: 4092–4101. 12821943 10.1038/sj.onc.1206598

[21] Meng Z, Lou Z, Liu Z, Li M, Zhao X, et al (2006) Crystal structure of human pyrroline-5-carboxylate reductase. J Mol Biol 359: 1364–1377. 16730026 10.1016/j.jmb.2006.04.053

[22] Nocek B, Chang C, Li H, Lezondra L, Holzle D, et al (2005) Crystal structures of delta1-pyrroline-5-carboxylate reductase from human pathogens Neisseria meningitides and Streptococcus pyogenes. J Mol Biol 354: 91–106. 16233902 10.1016/j.jmb.2005.08.036 PMC2792033

[23] Sander V, Reversade B, De Robertis EM (2007) The opposing homeobox genes Goosecoid and Vent1/2 self-regulate Xenopus patterning. EMBO J 26: 2955–2965. 17525737 10.1038/sj.emboj.7601705 PMC1894760

[24] Tian J, Ling L, Shboul M, Lee H, O′Connor B, et al (2010) Loss of CHSY1, a secreted FRINGE enzyme, causes syndromic brachydactyly in humans via increased NOTCH signaling. Am J Hum Genet 87: 768–778. 21129727 10.1016/j.ajhg.2010.11.005 PMC2997365

[25] Donald SP, Sun XY, Hu CA, Yu J, Mei JM, et al (2001) Proline oxidase, encoded by p53-induced gene-6, catalyzes the generation of proline-dependent reactive oxygen species. Cancer Res 61: 1810–1815. 11280728

[26] Hu CA, Donald SP, Yu J, Lin WW, Liu Z, et al (2007) Overexpression of proline oxidase induces proline-dependent and mitochondria-mediated apoptosis. Mol Cell Biochem 295: 85–92. 16874462 10.1007/s11010-006-9276-6

[27] Maxwell SA, Rivera A (2003) Proline oxidase induces apoptosis in tumor cells, and its expression is frequently absent or reduced in renal carcinomas. J Biol Chem 278: 9784–9789. 12514185 10.1074/jbc.M210012200

[28] Liu Y, Borchert GL, Surazynski A, Hu CA, Phang JM (2006) Proline oxidase activates both intrinsic and extrinsic pathways for apoptosis: the role of ROS/superoxides, NFAT and MEK/ERK signaling. Oncogene 25: 5640–5647. 16619034 10.1038/sj.onc.1209564

[29] Liu Y, Borchert GL, Donald SP, Diwan BA, Anver M, et al (2009) Proline oxidase functions as a mitochondrial tumor suppressor in human cancers. Cancer Res 69: 6414–6422. 19654292 10.1158/0008-5472.CAN-09-1223 PMC4287397

[30] Ernst T, Hergenhahn M, Kenzelmann M, Cohen CD, Bonrouhi M, et al (2002) Decrease and gain of gene expression are equally discriminatory markers for prostate carcinoma: a gene expression analysis on total and microdissected prostate tissue. Am J Pathol 160: 2169–2180. 12057920 10.1016/S0002-9440(10)61165-0 PMC1850818

[31] Kretz R, Bozorgmehr B, Kariminejad MH, Rohrbach M, Hausser I, et al (2011) Defect in proline synthesis: pyrroline-5-carboxylate reductase 1 deficiency leads to a complex clinical phenotype with collagen and elastin abnormalities. J Inherit Metab Dis 34: 731–739. 21487760 10.1007/s10545-011-9319-3

[32] Lee HX, Ambrosio AL, Reversade B, De Robertis EM (2006) Embryonic dorsal-ventral signaling: secreted frizzled-related proteins as inhibitors of tolloid proteinases. Cell 124: 147–159. 16413488 10.1016/j.cell.2005.12.018 PMC2486255

