# Supplementary figures and images for: Functional Specialization in Proline Biosynthesis of Melanoma
Source: PLoS One. 2012 Sep 14;7(9):e45190. doi: 10.1371/journal.pone.0045190 (PMC3443215; doi:10.1371/journal.pone.0045190)

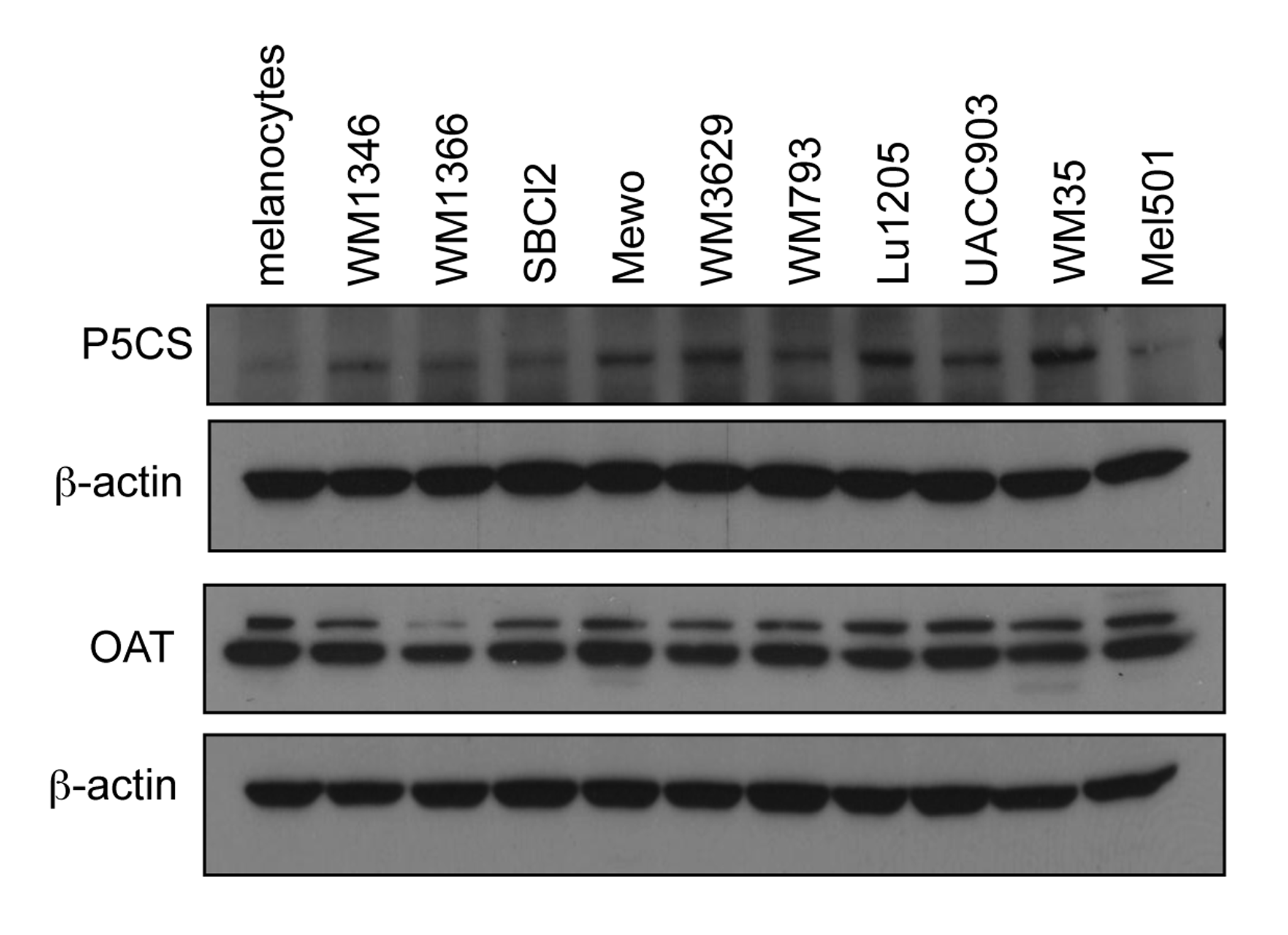

Supplement: Figure S1 — Melanoma cells express higher levels of P5CS but similar levels of OAT compared to primary melanocytes. Expression of P5CS and OAT in melanocytes relative to melanoma cells represented by a panel of ten cell lines was determined by Western blotting. Specificities of the antibodies used are indicated on the left of each panel. β-actin was used as loading control. (TIFF) [file pone.0045190.s001.tif]

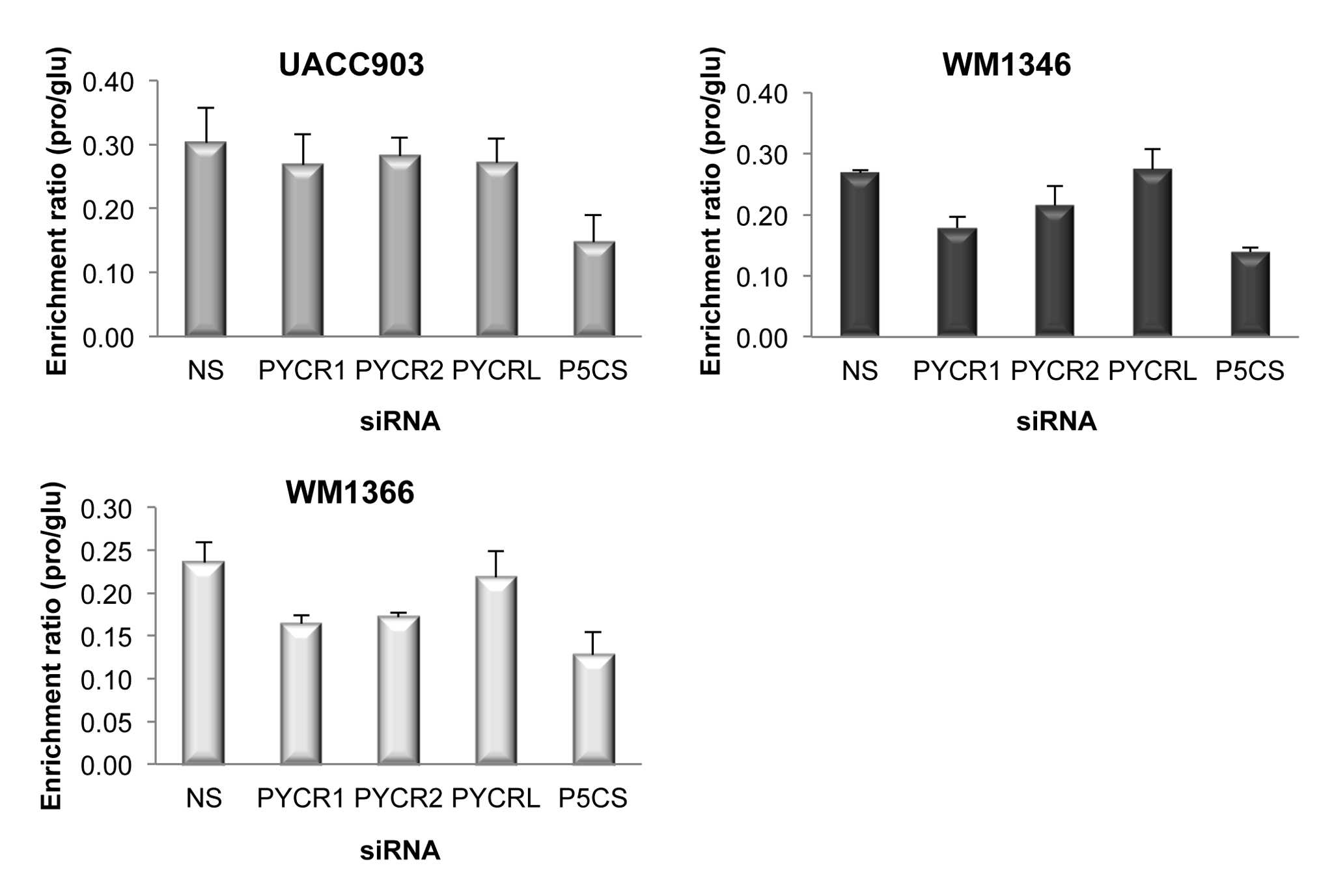

Supplement: Figure S2 — Effect of PYCRs silencing in UACC903, WM1346 and WM1366 cells. The production of proline from glutamate, expressed as the enrichment ratio (pro/glu), was measured in cells labeled for 8 hr with [U-13C] glutamine (1 mM) in the presence of 0.3 mM of proline in the medium. Error bars represent standard deviations of biological duplicate. (TIFF) [file pone.0045190.s002.tif]
