# Supplementary material for: Functional Specialization in Proline Biosynthesis of Melanoma
Source: PLoS One. 2012 Sep 14;7(9):e45190. doi: 10.1371/journal.pone.0045190 (PMC3443215; doi:10.1371/journal.pone.0045190)
Supplement: Table S1 — 13C enrichment in proline and glutamate in Lu1205 cells at different time points. Cells were fed with [U-13C] glutamine in the presence of 0.3 mM of exogenous proline. Measurements of isotopic enrichment in proline and glutamate were made 2, 4, 6 and 8 hr after labeling. Results represent technical duplicates and standard deviations are less than 10%. (DOCX) [file pone.0045190.s003.docx]

**Table S1.**

| **Time (h)** | **^13^C-enrichment in pro** | **^13^C-enrichment in glu** |
| --- | --- | --- |
| **2** | 0.10 | 0.32 |
| **4** | 0.09 | 0.30 |
| **6** | 0.11 | 0.33 |
| **8** | 0.09 | 0.32 |
