# Supplementary material for: Functional Specialization in Proline Biosynthesis of Melanoma
Source: PLoS One. 2012 Sep 14;7(9):e45190. doi: 10.1371/journal.pone.0045190 (PMC3443215; doi:10.1371/journal.pone.0045190)
Supplement: Table S2 — Knockdown of PYCRs is without effect on isotopic enrichment in TCA cycle metabolites. Lu1205 cells were fed with [U-13C] glutamine in the presence of 0.5 mM of exogenous proline and isotopic enrichment was calculated after 8 hr of labeling. Data represent average of biological duplicates and standard deviations are less than 5%. (DOCX) [file pone.0045190.s004.docx]

**Table S2.**

| **siRNA** | **^13^C enrichment in fumarate** | **^13^C enrichment in malate** | **^13^C enrichment in α-ketoglutarate** |
| --- | --- | --- | --- |
| **NS** | 0.28 | 0.28 | 0.30 |
| **PYCR1** | 0.24 | 0.25 | 0.31 |
| **PYCR2** | 0.29 | 0.30 | 0.32 |
| **PYCRL** | 0.24 | 0.25 | 0.27 |
| **P5CS** | 0.23 | 0.24 | 0.27 |
