# Supplementary material for: Functional Specialization in Proline Biosynthesis of Melanoma
Source: PLoS One. 2012 Sep 14;7(9):e45190. doi: 10.1371/journal.pone.0045190 (PMC3443215; doi:10.1371/journal.pone.0045190)
Supplement: Table S3 — 13C enrichment in proline in Lu1205 cells labeled with [U-13C] glutamine (8 h) in the presence of 0.5 mM of proline in the media. m0 is m/z 258 fragment ion, m1 is m/z 259, etc. Enrichment in proline is calculated with the following formula: Data represent average of two biological replicates and standard deviations are less than 5%. (DOCX) [file pone.0045190.s005.docx]

**Table S3.**

| **siRNA** | **Ion** | **Pro #1** | **Pro #2** | **Enrichment exp #1** | **Enrichment exp #2** | **Avg** |
| --- | --- | --- | --- | --- | --- | --- |
|  |  |  |  |  |  |  |
| **NS** | m0 | 0.889 | 0.882 |  |  |  |
|  | m1 | 0.015 | 0.021 |  |  |  |
|  | m2 | 0.016 | 0.021 |  |  |  |
|  | m3 | 0.007 | 0.003 |  |  |  |
|  | m4 | 0.077 | 0.072 | 0.09 | 0.09 | 0.09 |
| **PYCR1** | m0 | 0.921 | 0.919 |  |  |  |
|  | m1 | 0.009 | 0.011 |  |  |  |
|  | m2 | 0.023 | 0.015 |  |  |  |
|  | m3 | 0.004 | 0.005 |  |  |  |
|  | m4 | 0.046 | 0.051 | 0.06 | 0.06 | 0.06 |
| **PYCR2** | m0 | 0.929 | 0.924 |  |  |  |
|  | m1 | 0.003 | 0.012 |  |  |  |
|  | m2 | 0.015 | 0.011 |  |  |  |
|  | m3 | 0.002 | 0.004 |  |  |  |
|  | m4 | 0.054 | 0.052 | 0.06 | 0.06 | 0.06 |
| **PYCRL** | m0 | 0.849 | 0.846 |  |  |  |
|  | m1 | 0.012 | -0.002 |  |  |  |
|  | m2 | 0.033 | 0.044 |  |  |  |
|  | m3 | 0.003 | 0.005 |  |  |  |
|  | m4 | 0.106 | 0.113 | 0.13 | 0.14 | 0.13 |
| **P5CS** | m0 | 0.975 | 0.970 |  |  |  |
|  | m1 | 0.004 | 0.009 |  |  |  |
|  | m2 | 0.014 | 0.015 |  |  |  |
|  | m3 | 0.004 | 0.000 |  |  |  |
|  | m4 | 0.002 | 0.006 | 0.01 | 0.02 | 0.01 |
