# Supplementary material for: Functional Specialization in Proline Biosynthesis of Melanoma
Source: PLoS One. 2012 Sep 14;7(9):e45190. doi: 10.1371/journal.pone.0045190 (PMC3443215; doi:10.1371/journal.pone.0045190)
Supplement: Table S4 — 13C enrichment in glutamate in Lu1205 cells labeled with [U-13C] glutamine (8 h) in the presence of 0.5 mM of proline in the media. m0 is m/z 330 fragment ion, m1 is m/z 331, etc. Enrichment in glutamate is calculated with the following formula: Data represent average of two biological replicates and standard deviations are less that 5%. (DOCX) [file pone.0045190.s006.docx]

**Table S4.**

| **siRNA** | **Ion** | **Glu #1** | **Glu #2** | **Enrichment exp #1** | **Enrichment exp #2** | **Avg** |
| --- | --- | --- | --- | --- | --- | --- |
|  |  |  |  |  |  |  |
| **NS** | m0 | 0.644 | 0.655 |  |  |  |
|  | m1 | 0.011 | 0.014 |  |  |  |
|  | m2 | 0.061 | 0.060 |  |  |  |
|  | m3 | 0.013 | 0.009 |  |  |  |
|  | m4 | 0.286 | 0.278 | 0.33 | 0.32 | 0.32 |
| **PYCR1** | m0 | 0.671 | 0.653 |  |  |  |
|  | m1 | 0.032 | 0.021 |  |  |  |
|  | m2 | 0.069 | 0.081 |  |  |  |
|  | m3 | 0.010 | 0.009 |  |  |  |
|  | m4 | 0.230 | 0.248 | 0.28 | 0.30 | 0.29 |
| **PYCR2** | m0 | 0.668 | 0.641 |  |  |  |
|  | m1 | 0.002 | 0.001 |  |  |  |
|  | m2 | 0.062 | 0.061 |  |  |  |
|  | m3 | 0.011 | 0.011 |  |  |  |
|  | m4 | 0.272 | 0.303 | 0.31 | 0.34 | 0.33 |
| **PYCRL** | m0 | 0.703 | 0.684 |  |  |  |
|  | m1 | 0.011 | 0.020 |  |  |  |
|  | m2 | 0.060 | 0.058 |  |  |  |
|  | m3 | 0.010 | 0.011 |  |  |  |
|  | m4 | 0.229 | 0.244 | 0.27 | 0.29 | 0.28 |
| **P5CS** | m0 | 0.722 | 0.706 |  |  |  |
|  | m1 | 0.009 | 0.022 |  |  |  |
|  | m2 | 0.052 | 0.050 |  |  |  |
|  | m3 | 0.009 | 0.007 |  |  |  |
|  | m4 | 0.221 | 0.230 | 0.26 | 0.27 | 0.26 |
