# Supplementary material for: Functional Specialization in Proline Biosynthesis of Melanoma
Source: PLoS One. 2012 Sep 14;7(9):e45190. doi: 10.1371/journal.pone.0045190 (PMC3443215; doi:10.1371/journal.pone.0045190)
Supplement: Table S5 — Relative contribution of PYCRs to glutamate pathway. (A) Fraction of proline derived from glutamate expressed as the ratio of 13C enrichment of proline (product) over 13C enrichment of glutamate (precursor), measured upon silencing of PYCR1, PYCR2, PYCRL and P5CS. Lu1205 cells were labeled with [U-13C] glutamine (1 mM) for 8 h in the presence of 0.1, 0.3 and 0.5 mM of proline in the medium. (B) The same data are presented as % of change relative to non-specific siRNA (NS) control. Data are representative of two biological replicates and standard deviations are less than 5%. At 0.3 and 0.5 mM of exogenous proline, the data are consistent and show the same trend (as for PYCRL); at low concentration of proline (0.1 mM) there is not enough contribution of the salvage pathway to allow appreciable differences compared to the NS. (DOCX) [file pone.0045190.s007.docx]

**Table S5.**

**A**

|  | **Fraction of pro from glu (pro/glu)** | | |
| --- | --- | --- | --- |
| **Pro (mM)** | **0.1** | **0.3** | **0.5** |
| **NS_KD** | 0.76 | 0.46 | 0.29 |
| **PYCR1_KD** | 0.74 | 0.34 | 0.22 |
| **PYCR2_KD** | 0.78 | 0.38 | 0.20 |
| **PYCRL_KD** | 0.82 | 0.57 | 0.48 |
| **P5CS_KD** | 0.53 | 0.10 | 0.06 |
|  |  |  |  |

**B**

|  | **Change of enrichment of pro from glu (pro/glu) relative to NS (%)** | | |
| --- | --- | --- | --- |
| **Pro (mM)** | **0.1** | **0.3** | **0.5** |
| **NS_KD** | 0 | 0 | 0 |
| **PYCR1_KD** | -3 | -26 | -24 |
| **PYCR2_KD** | 3 | -17 | -31 |
| **PYCRL_KD** | 8 | 24 | 66 |
| **P5CS_KD** | -30 | -78 | -79 |
