# Supplementary material for: Functional Specialization in Proline Biosynthesis of Melanoma
Source: PLoS One. 2012 Sep 14;7(9):e45190. doi: 10.1371/journal.pone.0045190 (PMC3443215; doi:10.1371/journal.pone.0045190)
Supplement: Table S6 — Relative contribution of PYCRs to ornithine pathway. (A) Fraction of proline derived from ornithine expressed as the ratio of 13C enrichment of proline (product) over 13C enrichment of ornithine (precursor), measured upon silencing of PYCR1, PYCR2, PYCRL and P5CS. Lu1205 cells were labeled with [U-13C] ornithine (1 mM) for 8 h in the absence of proline in the medium. (B) The same data are presented as % of change relative to non-specific siRNA (NS) control. Data are representative of two biological replicates and standard deviations are less than 5%. (DOCX) [file pone.0045190.s008.docx]

**Table 6S.**

**A B**

|  | **Fraction of pro from orn (pro/orn)** |
| --- | --- |
| **Pro (mM)** | **0** |
| **NS_KD** | 0.36 |
| **PYCR1_KD** | 0.19 |
| **PYCR2_KD** | 0.40 |
| **PYCRL_KD** | 0.25 |
| **P5CS_KD** | 0.48 |

|  | **Fraction of pro from orn (pro/orn) relative to NS (%)** |
| --- | --- |
| **Pro (mM)** | **0** |
| **NS_KD** | 0 |
| **PYCR1_KD** | -47 |
| **PYCR2_KD** | 11 |
| **PYCRL_KD** | -31 |
| **P5CS_KD** | 33 |
