# Supplementary material for: Functional Specialization in Proline Biosynthesis of Melanoma
Source: PLoS One. 2012 Sep 14;7(9):e45190. doi: 10.1371/journal.pone.0045190 (PMC3443215; doi:10.1371/journal.pone.0045190)
Supplement: Table S7 — Kinetic characterization of recombinant PYCRs. Apparent kinetic parameters of human PYCRs with respect to substrate (A) and cofactors (B) were determined by monitoring the turnover of co-factors (NADH or NADPH). Results from steady-state kinetics experiments, obtained at saturating concentrations of cofactors*** and substrate*** (P5C) were graphed and fit with non-linear regression**. Apparent catalytic efficiencies of human PYCRs with respect to P5C and cofactors (C) were calculated using the apparent kcat and Km values presented in A and B. Apparent constants of PYCR inhibition by proline at saturating concentrations of co-factors (D) (1 mM NADH for PYCR1 and PYCR2 and 1.25 mM NADPH for PYCRL) were determined by fitting the steady-state kinetics data of P5C conversion to proline at varying concentrations of inhibitor with competitive inhibition equation. (DOCX) [file pone.0045190.s009.docx]

**Table S7.**

**A**

| **Enzyme** | **P5C** | | | |
| --- | --- | --- | --- | --- |
|  | **Km_app_ (mM)*** | | **kcat_app_ (s^-1^)*** | |
|  | **NADH** | **NADPH** | **NADH** | **NADPH** |
| **PYCR1** | 1.72 + 0.15 | 2.15 + 0.09 | 70.4 + 2.0 | 29.4 + 0.5 |
| **PYCR2** | 1.00 + 0.04 | 1.70 + 0.07 | 149.0 + 1.7 | 85.0 + 1.2 |
| **PYCRL** | 4.64 + 0.49 | 0.38 + 0.07 | 197.0 + 9.7 | 35.0 + 1.1 |

**B**

| **Enzyme** | **Co-factor** | | | |
| --- | --- | --- | --- | --- |
|  | **Km_app_ (mM)*** | | **kcat_app_ (s^-1^)*** | |
|  | **NADH** | **NADPH** | **NADH** | **NADPH** |
| **PYCR1** | 0.26 + 0.02 | 1.20 + 0.16 | 63.9 + 1.5 | 45.6 + 4.0 |
| **PYCR2** | 0.22 + 0.04 | 0.24 + 0.04 | 218.8 + 13.0 | 93.2 + 4.7 |
| **PYCRL** | 0.42 + 0.04 | 0.37+ 0.05 | 196.4 + 6.0 | 24.9 + 1.0 |

**C**

| **Enzyme** | **kcat_app_/Km_app_ (M^-1^s^-1^) x 10^3^** | | | |
| --- | --- | --- | --- | --- |
|  | **P5C** | | **NADH** | **NADPH** |
|  | **NADH** | **NADPH** |  |  |
| **PYCR1** | 40.9 + 3.8 | 13.7 + 0.6 | 249.6 + 19.8 | 38.0 + 6.1 |
| **PYCR2** | 148.1 + 6.2 | 50.1 + 2.2 | 1000.3 + 190.2 | 388.3 + 67.6 |
| **PYCRL** | 42.4 + 4.9 | 92.5 + 17.2 | 467.6 + 46.8 | 67.3 + 9.5 |

**D**

| **Proline** | |
| --- | --- |
| **Enzyme** | **Ki_app_ (mM)*** |
| **PYCR1** | 0.589 + 0.067 |
| **PYCR2** | 0.096 + 0.011 |
| **PYCRL** | 8.474 + 0.97 |

*Data shown represent best-fit values of 3 determinations performed at different enzyme concentrations as described in Materials and Methods.

**PYCR1 and 2 data with respect to substrate were fit with Michaelis-Menten equation. PYCRL data with respect to substrate were fit with allosteric sigmoidal model equation. Data with respect to cofactors were fit with Michaleis-Menetn equation for all PYCRs, except PYCR1 and 2 in the presence of NADPH and NADH, respectively, where substrate inhibition was observed and taken into account.

***Concentrations of cofactors used: PYCR1: 2.5 mM NADH or NADPH, PYCR2: 1.25 mM NADH and 0.625 mM NADPH, PYCRL: 5 mM NADH and 5 mM NADPH. P5C was used at 10 mM for all PYCRs.
